# Sex Related Differences in the Complex Relationship between Coffee, Caffeine and Atrial Fibrillation

**DOI:** 10.3390/nu15153299

**Published:** 2023-07-25

**Authors:** Francesca Coppi, Valentina Bucciarelli, Giorgia Sinigaglia, Giada Zanini, Valentina Selleri, Milena Nasi, Marcello Pinti, Sabina Gallina, Anna Vittoria Mattioli

**Affiliations:** 1 Department of Medical and Surgical Sciences for Children and Adults, University of Modena and Reggio Emilia, 41124 Modena, Italy; francesca.coppi@unimore.it; 2 Cardiovascular Sciences Department, Azienda Ospedaliero—Universitaria delle Marche, 60126 Ancona, Italy; 3 Department of Life Sciences, University of Modena and Reggio Emilia, 41125 Modena, Italygiada.zanini@unimore.it (G.Z.); valentina.selleri@unimore.it (V.S.); marcello.pinti@unimore.it (M.P.); 4 Surgical, Medical and Dental Department of Morphological Sciences Related to Transplant, Oncology and Regenerative Medicine, University of Modena and Reggio Emilia, 41125 Modena, Italy; milena.nasi@unimore.it; 5 Department of Neuroscience, Imaging and Clinical Sciences, University of Chieti-Pescara, 66100 Chieti, Italy; sabina.gallina@unich.it

**Keywords:** women, caffeine, coffee, energy drinks, atrial fibrillation

## Abstract

This literature review aims to explore the data of articles published on the association between coffee, caffeine and atrial fibrillation and to analyze any differences between the two sexes. Several factors influence this complex relationship; genetic, environmental and psychosocial factors come into play in the pathophysiology of atrial fibrillation. These factors are expressed differently in women and men. However, the analysis of the literature has shown that comparison works between the two sexes are extremely rare. Most population-based and prospective studies either analyze aggregated data or focus on exclusively male or female populations. This results in a lack of information that could be useful in the prevention of and treatment approach to atrial fibrillation. It is necessary to deepen this issue with dedicated studies.

## 1. Introduction

There have been conflicting results on the association between coffee, caffeine and atrial fibrillation (AF), and studies focusing on the influence of sex on this complex relationship are very rare [1,2,3,4,5].

The widespread diffusion of coffee and tea among adults and of energy drinks among young people make it necessary to broaden our knowledge to understand the mechanisms underlying the potential pro-arrhythmic role of caffeine-containing drinks [1,2,3,4,5]. This literature review aims to explore the data of articles published on the topic and to analyze any differences between the two sexes that have emerged from scientific studies.

Coffee is an extremely widespread drink and is part of the culture of most people [6]. It is included in most diets because it has been shown to have favorable effects on cardiovascular health [7]. These effects depend on the different substances that coffee contains [8]. Caffeine content is the aspect most analyzed in the literature due to the fear that the effects on health could be deleterious [7,8]. Caffeine is an alkaloid found in coffee beans and is also present in other dietary sources such as tea, soft and energy drinks, chocolate and cocoa beverages. The caffeine content varies, with coffee typically containing 4 to 180 mg/150 mL, cola 15 to 29 mg/180 mL, tea 24 to 50 mg/150 mL, cocoa 2 to 7 mg/150 mL, and chocolate 1 to 36 mg/28 mg. Energy drinks can range from 100 to 286 mg of caffeine per serving, although some brands may contain as much as 550 mg per can or bottle. The caffeine content in one cup of coffee varies according to the geographical region and also depends on the method of preparation. In Northern Europe and the United Kingdom, a cup of coffee contains 140 mg of caffeine, in the USA 85 mg of caffeine and in Southern Europe, where espresso and mocha are common, the amount of caffeine is around 50 mg [3,6,9].

The Food Standards Authority of Australia and New Zealand has defined three levels of caffeine intake: low, moderate and high [10] (Table 1).

These guidelines provide a range of caffeine intake levels based on body weight, with the assumption that a 70 kg weight adult is being considered.

No differences between women and men are highlighted except for a reference to pregnancy and breastfeeding.

The Food Standards Code limits the amount of caffeine that can be added to cola-type soft drinks and energy drinks. Foods containing added caffeine must also have a statement on the label that the product contains caffeine. It is important to indicate the quantity of guarana on the drink label due to the molecular similarity with caffeine. [10].

In cola soda-type drinks, the total caffeine content should not exceed 145 mg/kg. Energy drinks are regulated by Standard 2.6.4 [11] of the Code which defines the maximum permitted levels of caffeine and other substances in these drinks (the maximum amount of caffeine is 320 mg per liter). Furthermore, additional labeling requirements insist that warning are included. It advises that products are not suitable for young children, pregnant or nursing women and caffeine-sensitive individuals [10].

In 2015, the European Food Safety Authority (ESFA) published a “Scientific Opinion on the safety of caffeine” suggesting that single doses of caffeine up to 200 mg (about 3 mg/kg body weight for a 70 kg adult) do not give rise to safety problems (Table 2). The same amount does not give rise to safety concerns if consumed <2 h before strenuous exercise performed under normal environmental conditions. The ESFA also points out other components of “energy drinks” at the concentrations declared in such drinks (approximately 300–320, 4000 and 2400 mg/L of caffeine, taurine and d-glucurono-γ-lactone, respectively). The intake of alcohol at doses up to about 0.65 g/kg body weight does not affect the safety of single doses of caffeine up to 200 mg.

There are differences between habitual and non-habitual coffee consumers. Similarly, in pregnant women, the habitual consumption of caffeine up to 200 mg per day does not give rise to safety concerns. Single doses of caffeine and usual caffeine intakes of up to 200 mg consumed by breastfeeding women do not raise safety concerns for infants. For children and adolescents, the information available to date is insufficient to derive a safe intake of caffeine. However, the diffusion of the habit of introducing soft drinks containing caffeine is gradually increasing in younger people and it will be necessary to acquire further information. The panel considers that the identified acute dose of harmless caffeine for adults (3 mg/kg body weight per day) could be used for calculating the daily intake of caffeine of no concern for these individuals [12]. No differences between women and men are highlighted except for a reference to pregnancy.

## 2. Caffeine

The half-life of caffeine can vary in a healthy adult and typically ranges from 2.5 to 10 h. Habitual consumption, the long-term consumption of caffeine and the consumption of large amounts may prolong the half-life further [13]. After consuming caffeine, the maximum plasma level is typically reached after around 2 h. However, plasmatic levels start to increase as early as 30 min after consumption [6,9]. Plasmatic levels can be influenced by various factors, including the simultaneous consumption of food [14].

Considering these factors, it is advisable to be mindful of caffeine intake, especially when consuming larger amounts or on a long-term basis. Monitoring one’s caffeine intake and adhering to the recommended guidelines can help individuals maintain a balanced and healthy approach to caffeine consumption [15].

The creation of validated questionnaires for assessing caffeine intake in specific populations is crucial for accurate evaluation. Since caffeine is present in various drinks and foods, including coffee and energy drinks, it is important to have reliable tools to measure and quantify caffeine consumption.

Coffee is indeed a widely consumed source of caffeine among adults, while energy drinks are often favored by young people. The preparation method of coffee can significantly impact the amount of caffeine extracted and present in the final drink. Factors such as brewing time, water temperature, bean type, and coffee-to-water ratio can all affect caffeine content [14,16,17,18,19].

This variability in caffeine levels can introduce bias when assessing overall caffeine intake from coffee. To address this issue, validated questionnaires often include specific questions about coffee-preparation methods, such as the type of coffee consumed (e.g., espresso, drip coffee), the frequency of consumption, and the average serving size. These details help researchers or healthcare professionals obtain a more accurate estimate of caffeine intake from coffee and account for any biases related to preparation methods [16,17,18,19].

An analysis of UK Biobank data aims to assess associations between coffee subtypes on incident cardiovascular outcomes [20]. Coffee subtypes were defined as decaffeinated, ground, and instant, then categorized as 0, <1, 1, 2–3, 4–5, and >5 cups/day, and compared to non-drinkers [20].

Consumption of ground and instant coffee was associated with a significant reduction in arrhythmia at 1–5 cups per day, whereas this finding was not observed in subjects consuming decaffeinated coffee. The lowest reported risk was 4–5 cups/day for ground coffee [HR 0.83, confidence interval (CI) 0.76–0.91, *p* < 0.0001], whereas it was 2–3 cups/day for instant coffee (HR 0.88, CI 0.85–0.92, *p* < 0.0001). All coffee subtypes were associated with a reduction in CVD incidence (the lowest risk was 2 to 3 cups/day for decaf, *p* = 0.0093; ground, *p* < 0.0001; and instant coffee, *p* < 0.0001) compared to non-drinkers [20].

Furthermore, the questionnaire may include specific questions about the brand, size and frequency of consumption of energy drinks and caffeinated beverages in order to assess caffeine intake accurately. By using validated questionnaires tailored to the specific population and taking into account the various sources of caffeine, including their preparation methods, researchers and healthcare professionals can obtain more reliable data on caffeine intake and its potential effects on health [15,21].

When analyzing the metabolism of caffeine, it emerges that more than 95% of caffeine is metabolized by cytochrome P450 1A2 (CYP1A2). Studies have reported that a common polymorphism in the CYP1A2 gene, the rs762551 variant, is able to modify caffeine metabolism by reducing enzyme activity and inducibility [22]. Specifically, individuals with the AC and CC genotypes of this polymorphism are often referred to as “slow metabolizers,” while those with the AA genotype are defined as “fast metabolizers” [22].

Slow metabolizers may have reduced activity of the CYP1A2 enzyme, resulting in a slower breakdown of caffeine in the body. On the other hand, fast metabolizers with the AA genotype have a more efficient CYP1A2 enzyme, leading to faster caffeine metabolism [22].

The association between coffee intake and the risk of hypertension, ischemic heart disease and impaired fasting glucose can be modified by this polymorphism in a dose-dependent manner.

For example, some studies have suggested that slow metabolizers (AC or CC genotype) may be more sensitive to the potential adverse effects of high coffee consumption, such as an increased risk of myocardial infarction or hypertension [22,23,24,25].

There are genetic differences between the sexes that can influence the metabolism effects of caffeine. The amount of CYP1A2 in males appears to be higher than in females. In the clinical evaluation of the subjects, it is also necessary to include smoking, because CYP1A2 expression is highly inducible by smoking [26,27,28]. In a recent study, we evaluated the changes in coffee consumption that occurred during the pandemic in a group of adult women and compared the changes in smoking versus non-smoking women [28]. Phenolic compounds are present in espresso coffee and are metabolized in the body differently in women than in men. Several studies reported that the activity of CYP2B6, CYP2A6 and CYP3A is higher in women than men. On the contrary, the activity of CYP2D6, CYP2E1 and CYP1A2 is a little higher in men [28,29]. Similarly, the effects of gallic acid, caffeic acid, and quercetin on CYP1A, CYP2A, CYP2E1, and CYP3A are influenced by sex [29,30]. The study reported an increase in coffee intake in smokers compared to non-smoking women [28]. This increase in coffee consumption led to a greater dose of antioxidants from espresso counteracting some negative effects on health of smoking. These effects depend on the dosage and bioavailability of nutrients. During the pandemic, several studies reported an increase in sugar-rich and fat-rich foods, the so-called “comfort food”, to cope with stress [31].

The interactions between coffee intake, genetic variations and health outcomes can be complex and influenced by various factors [32]. Further research is needed to fully understand the implications of this polymorphism and its influence on the associations between coffee consumption and specific health conditions.

### Molecular and Cellular Action of Caffeine

Caffeine acts as a non-selective competitive antagonist of adenosine receptors, specifically the A1 and A2A subtypes. By blocking these receptors, caffeine inhibits the effects of adenosine, a neuromodulator that promotes relaxation and sleep. This antagonistic action contributes to caffeine’s stimulating effects, promoting mental alertness, wakefulness and reducing fatigue [33].

Mechanisms of action of caffeine include: inhibition of phosphodiesterase enzyme, antagonism of adenosine receptors and activation of ryanodine receptors.

- Inhibition of phosphodiesterase enzyme: Caffeine inhibits the activity of phosphodiesterase enzymes, which normally break down cyclic adenosine monophosphate (cAMP) and cyclic guanosine monophosphate (cGMP). By inhibiting phosphodiesterase, caffeine increases the levels of cAMP and cGMP, leading to various physiological effects [34].
- Antagonism of adenosine receptors: Caffeine acts as a competitive antagonist of adenosine receptors, specifically the A1 and A2A subtypes. By binding to these receptors, caffeine blocks the actions of adenosine, a neuromodulator that promotes relaxation and sleep. This antagonistic action of caffeine contributes to its stimulating effects, such as increased alertness and wakefulness [33].
- Activation of ryanodine receptors: Caffeine also has the ability to activate ryanodine receptors, which are involved in the release of calcium from intracellular stores, such as the sarcoplasmic reticulum. This release of calcium can have effects on various organs and systems in the body [35,36].

These mechanisms of action contribute to the wide-ranging effects of caffeine on multiple organs and physiological processes. However, it is important to note that the specific effects of caffeine can vary among individuals, depending on factors such as tolerance, sensitivity and overall health.

The stimulating action of caffeine on the heart leads to an increased heart rate and contractility, which can be beneficial in certain situations. It can enhance cardiac output and improve blood flow to vital organs. Additionally, caffeine has been reported to cause coronary vasodilation, which can improve blood flow through the coronary arteries and potentially benefit individuals with coronary artery disease [37].

However, it is important to note that excessive caffeine consumption or sensitivity to its effects can have negative consequences. The vasoconstrictive effects of caffeine on cerebral blood vessels can lead to a decrease in flow to the brain. While this effect can be useful in relieving headaches or migraines for some individuals, it may be dangerous if the overall flow to the brain is compromised [38].

Furthermore, the effects of caffeine can vary depending on an individual’s sensitivity, tolerance, and overall health. Some individuals may be more susceptible to the cardiovascular effects of caffeine, such as increased heart rate or arrhythmias, while others may experience minimal or no adverse effects [9,25].

## 3. Effects of Coffee and Caffeine Linked to Arrhythmias Development

The relationship between caffeine consumption and atrial fibrillation has been a topic of study and debate in medical research. A major contributor to cardiac arrhythmias, including AF, is played by modifiable risk factors such as obesity, unhealthy diet, lack of physical activity, sedentary lifestyle, smoking, alcohol abuse, and hypertension [39]. Some studies have suggested a potential association between caffeine intake and AF, while others have found no significant link. The findings have been somewhat conflicting, and more research is needed to establish a clear and definitive relationship [39,40,41,42,43,44].

**Figure 1:**
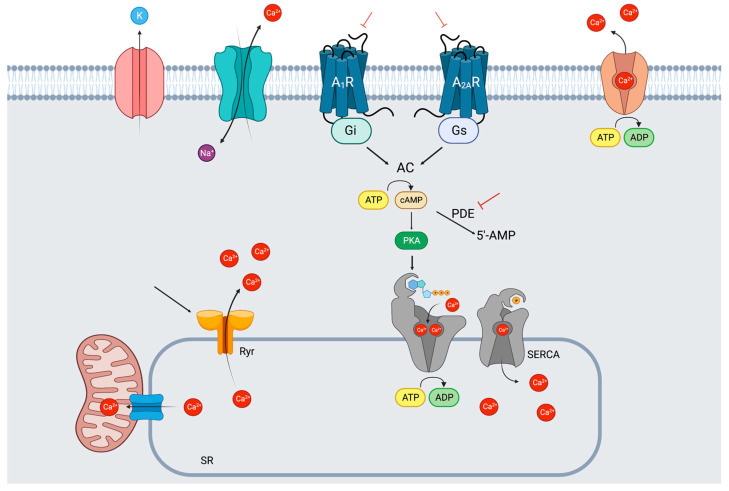
Caffeine has a range of effects on sympathetic activation, intracellular calcium trafficking and adenosine receptors. Caffeine can have a dual effect on cyclic adenosine monophosphate (cAMP) intracellular levels. On one hand, caffeine inhibits phosphodiesterase 2 (PDE2) by increasing cAMP and cytosolic [Ca++] by blocking calcium reuptake into the sarcoplasmic reticulum (SR) mediate by Sarco-Endoplasmic Reticulum Calcium ATPase (SERCA). On the other hand, caffeine inhibits adenosine receptors (AR) so reducing cAMP levels. Furthermore, caffeine activates ryanodine receptors (RyR), thus contributing to calcium release from the SR. The increase in intracellular [Ca++] can induce atrial arrhythmia by improving the automaticity of atrial pacemaker cells. Conversely, the inhibition of adenosine receptors A1R and A2AR confers antiarrhythmic properties to caffeine, as adenosine shortens atrial refractoriness.

Caffeine, as a stimulant, can have various effects on the cardiovascular system, including increased blood pressure and heart rate. It is known to temporarily block adenosine receptors, which may have implications for cardiac electrical activity. Adenosine is involved in regulating the heart’s electrical signals, and the blocking of its receptors by caffeine could potentially affect the heart’s rhythm [45]. The underlying mechanisms and triggering factors of AF are not yet fully understood. However, some studies have suggested that the adenosine-mediated signaling pathway, which can be influenced by caffeine, may contribute to the initiation of AF. This pathway could lead to increased spontaneous sarcoplasmic reticulum calcium release in atrial myocytes, potentially playing a role in the development of AF. As shown in Figure 1, caffeine’s effects on AF may be mediated through neurohormonal stimulation and sympathetic activation, particularly in individuals who are not habitual coffee drinkers. It is suggested that caffeine may enhance these effects in nonhabitual consumers [46,47,48]. In cases of caffeine toxicity, which usually occur at very high doses, supraventricular tachycardia, atrial fibrillation and ventricular fibrillation can occur. It is important to note that such toxic effects are rare and typically associated with excessive consumption or sensitivity to caffeine [49,50].

## 4. Effects of Coffee and Caffeine Linked to Arrhythmias Prevention

There is ongoing debate and research on the potential relationship between caffeine and coffee consumption and their effects on arrhythmic risk factors and cardiovascular health. The question of whether caffeine and coffee are arrhythmic risk factors is still open; on the contrary, some longitudinal studies have suggested a protective effect of coffee on cardiovascular risk. Additionally, a recent study analyzed the effects of coffee consumption on arterial hypertension and found that regular coffee drinking is associated with lower systolic blood pressure, pulse pressure, aortic blood pressure and aortic pulse pressure, but with similar arterial stiffness [51,52].

The beneficial effects of coffee could be attributed to the presence of various substances with antioxidant and anti-inflammatory properties. These substances may contribute to the potential protective effects observed in some studies [1,6].

Coffee is a complex beverage containing numerous chemical compounds, and our understanding of its biological properties is continually evolving as research progresses. While some compounds in coffee have been well-studied, many others may not have received as much attention or may still be unidentified.

Caffeine, chlorogenic acid, trigonelline, cafestol, kahweol and ferulic acid are among the better-described constituents of coffee that have been studied for their potential effects on health.

Chlorogenic acid (CGA) is a type of polyphenol known for its antioxidant properties. It may play a role in reducing oxidative stress in the body [53,54,55]. CGA reduces systemic inflammation and oxidative stress. Through their action on oxidative stress, CGAs improve endothelial function and increase the bioavailability of nitric oxide, with a consequent reduction in blood pressure in subjects who habitually consume coffee [56]. Caffeine and CGAs act on lipid synthesis and fatty acid metabolism through different pathways [57]. A well-studied pathway is the suppression of lipid synthesis enzyme activity. Both caffeine and CGAs can suppress the activity of acetyl-CoA carboxylase, fatty acid synthase and stearoyl-CoA desaturase. By inhibiting these enzymes, caffeine and CGAs can potentially reduce the production of fatty acids [58,59].

Furthermore, caffeine can increase fatty-acid beta-oxidation by stimulating the activity of carnitine palmitoyltransferase, an enzyme that facilitates the transport of fatty acids into mitochondria for the oxidation process. This can lead to the utilization of fatty acids as an energy source [60,61]. In addition, caffeine and CGAs have been found to activate peroxisome proliferator-activated receptor alpha (PPAR-alpha) in the liver and adipose tissues. PPAR-alpha is a transcription factor that regulates lipid metabolism. The activation of PPAR-alpha can lead to increased fatty acid oxidation and decreased lipogenesis (the process of synthesizing new fatty acids) [62,63]. Finally, CGAs alone have been shown to downregulate sterol regulatory element-binding protein-1C (SREBP-1C). SREBP-1C is a transcription factor that promotes the synthesis of fatty acids and triglycerides. By inhibiting SREBP-1C, CGAs can help control lipogenesis and reduce the production of new fatty acids. The effects of caffeine and CGAs on lipid metabolism may vary depending on the specific concentrations used, the duration of exposure and the experimental models or systems employed in the studies. Further research is needed to fully understand the extent and clinical implications of these effects on human lipid metabolism [64].

Ferulic acid is another polyphenol present in coffee with antioxidant and anti-inflammatory properties [65]. Ferulic acid can be found in cereals, fruits and vegetables [66]. Ferulic acid exhibits a vasodilatory effect related to its ability to improve the bioavailability of nitric oxide (NO), which exerts effects on platelet aggregation, blood pressure, and leukocyte adhesion [67]. Cafestol and Kahweol are diterpenes found in coffee oils, and they have been the subject of significant research and discussion due to their potential effects on cholesterol levels [68]. On the one hand, cafestol and kahweol have been associated with raising LDL cholesterol (low-density lipoprotein cholesterol) [69]. Early studies suggested that coffee diterpenes (particularly cafestol), effectively increase human plasma triacylglycerol and low-density lipoprotein (LDL), may be a potential risk of inducing cardiovascular disease [69]. However, from a fuller perspective, cafestol and kahweol exhibit a two-faced effect. In addition to the deleterious effects on serum lipid levels and liver enzymes in some cases, extensive studies have demonstrated that cafestol and kahweol exhibit a wide variety of pharmacological activities, including anti-inflammatory, anti-angiogenic and anti-tumoral effects [49,70]. The cholesterol-raising effect of cafestol and kahweol is primarily observed when coffee is prepared using methods that do not involve a paper filter, such as French press or espresso. Paper filters effectively trap these compounds, reducing their presence in the final cup. Therefore, filtered coffee, like that prepared with a drip coffee maker, tends to have a smaller impact on cholesterol levels compared to unfiltered coffee [6,9].

Trigonelline is a natural alkaloid found in various plants, including coffee beans. It is a water-soluble compound that contributes to the unique flavor and aroma of coffee [49]. Trigonelline is also present in other sources such as fenugreek seeds and certain legumes. Trigonelline is biosynthesized from nicotinic acid (vitamin B3) and is considered a derivative of this vitamin. During the roasting process of coffee beans, trigonelline breaks down to form other compounds, including nicotinic acid (niacin) and other volatile aroma compounds, which play a role in the overall taste and aroma of coffee. Trigonelline may exhibit antioxidant properties, helping to combat oxidative stress in the body. Some research suggests that it could have protective effects against certain chronic diseases, though more studies are needed to establish specific health benefits [71]. It is worth noting that trigonelline is distinct from caffeine, another well-known compound found in coffee, although both contribute to the overall properties of the beverage. As with many natural compounds, the full extent of trigonelline’s effects on health and well-being is an area of ongoing research.

## 5. Clinical Studies on Coffee and Caffeine and Atrial Fibrillation

A very recent study analyzed data from 449,563 participants in the UK Biobank, with no cardiovascular disease at the time of enrollment (55.3% were women). The mean follow-up time was 12.5 years. As previously reported, drinking 4 to 5 cups/day of ground coffee and 2 to 3 cups/day of instant coffee reduced incident arrhythmias [20]. Subjects who are accustomed to drinking decaffeinated coffee did not show this protective effect on arrhythmias suggesting that caffeine does not have a proarrhythmic effect. It should be noted that interference from one’s lifestyle and other confounding factors such as age and gender is likely to exist. The study then analyzed the effects of different coffee subtypes on all-cause mortality and cardiovascular mortality and found significant reductions in both: all-cause mortality (HR 0.86, CI [0.83–0.89], *p* < 0.0001) and CV mortality (HR 0.82, CI [0.74–0.90], *p* < 0.0001). Incident CVD risk was reduced in subjects who habitually drank up to 5 cups of coffee per day with no differences across all coffee subtypes [40]. This very recent evidence is in line with other previous studies. The Danish Diet, Cancer and Health study evaluated the association between the daily amount of caffeine and the risk of atrial fibrillation or flutter. Data were collected through a detailed, semiquantitative food-frequency questionnaire (FFQ). The questionnaire assessed caffeine intake by summing the daily intake from coffee, tea, cola soda, cocoa and chocolate for each subject. The main source of caffeine was coffee, a drink widely consumed by adults in Denmark. Atrial fibrillation or flutter was reported in 555 subjects (373 men and 182 women) during the follow-up period. The authors used the lowest quintile of caffeine consumption as a reference, data are shown in Table 1. The authors concluded that they did not find any risk of atrial fibrillation or flutter associated with caffeine consumption [10]. In the Danish Diet, Cancer and Health study, the number of women enrolled was slightly higher than the number of men. Apparently, the two sexes are balanced; however, it is well known that in order to be able to effectively evaluate whether there is a difference due to sex, it is necessary to include this endpoint in the study and carry out an adequate recruitment comparing the data of the two groups. Indeed, the comparison between sexes was not performed [72]. In the Multifactor Primary Prevention Study, the consumption of 1–4 cups of coffee per day was associated with an increased risk of developing atrial fibrillation (OR 1.24; 95% CI: 1.00, −1.54); on the contrary, the consumption of >4 cups of coffee was not associated with a risk of atrial fibrillation [73].

Some studies have analyzed exclusively female populations. The Women Health study examined the relationship between caffeine consumption and incident AF in 33,638 middle-aged women taking different levels of caffeine. Caffeine intake was assessed using an FF questionnaire that evaluated the average consumption during the previous year of caffeine-containing foods and beverages. Coffee, tea, decaffeinated coffee and types of caffeinated and decaffeinated cola soda, were also included in the evaluation. The main source of caffeine intake was coffee (81.3%), followed by tea (10.0%), a minor contribution was identified from other sources: low-calorie cola with caffeine (5.6%), cola with caffeine (1.2%) and chocolate (0.3%). Age-adjusted Cox proportional hazard models found no increased risk of AF in women who consumed large amounts of caffeine (*p* for linear trend: 0.44). The results suggest a U-shaped association with the lowest risk seen in women in the third quintile of caffeine intake (median: 285 mg/d) [74].

When analyzing a group of 600 atrial fibrillation patients in order to identify the role of caffeine on spontaneous cardioversion, we found that non-habitual and low coffee drinkers showed the highest probability of spontaneous conversion (OR 1.93 95% CI 0.88–3.23; *p* = 0.001). In the group of subjects with arterial hypertension, moderate but not high coffee consumption had the lowest probability of spontaneous conversion (OR 1.13 95% CI 0.67–1.99; *p* = 0.05) suggesting that in these patients, other variables take place [48]. In patients with arterial hypertension, it is possible that atrial remodeling contributes to the onset of AF and delayed cardioversion of the arrhythmia [75].

Mendelian randomized studies showed no association between coffee consumption and the risk of AF. The Atrial Fibrillation Consortium study, a genome-wide association study that included 375,833 individuals, analyzed nine single nucleotide polymorphisms associated with coffee consumption. The odds ratio of AF for a genetically predicted 50% increase in coffee consumption was 0.98 (95% confidence interval, 0.88, 1.10; *p* = n.s.). Furthermore, analyses that separated coffee-related single-nucleotide polymorphisms based on their association with blood levels of caffeine metabolites revealed no association with AF [76].

The Physicians’ Health Study evaluated the association between coffee consumption and atrial fibrillation risk in an all-male population. Data were collected using self-reported food frequency questionnaires. The hazard ratios (95% CI) of AF were 1.0 for rare/no coffee consumption, and was used as a reference). HR for categories of coffee consumption is reported in Table 1. The results of this study suggest a lower risk of AF among men who consumed 1 to 3 cups of coffee per day [77].

A prospective study analyzed 1475 participants and followed up for 12 years. The most common source of caffeine was coffee (89.1%) followed by tea in 10.2%. Smokers (more than one cigarette/day) were 15.5%. The study found that caffeine consumption >320 mg/day significantly reduced the risk of AF, regardless of CYP1A2 polymorphism (*p* = 0.008) [78].

In a study of 400 patients (51.2% males), the risk of developing AF was associated with recent stress, increased recent coffee consumption and obesity. Acute stress can lead to changes in lifestyle and dietary habits including increased coffee and tea consumption. Increased coffee consumption was more significant in nonhabitual drinkers, resulting in a higher risk of developing AF [OR 4.1; I95% (CI): 1.98–4.56; *p* < 0.001]. When analyzing the factors that influenced the spontaneous cardioversion of the arrhythmia, it emerged that AF that appeared following recent acute stress had the highest probability of spontaneous conversion. In this case, the combined adrenergic action of the two triggers can favor the appearance of a transient arrhythmia [79]. Stress and anxiety are triggers for the development of arrhythmias, and at the same time, indicate a series of lifestyle changes including eating habits and diet [80,81]. Non-habitual coffee drinkers may experience caffeine as a trigger for arrhythmias. This phenomenon was also reported during the recent pandemic which led to important lifestyle changes in the population [28,82,83,84]. Table 3 shows data from perspective and cohort studies.

## 6. Limitations

The major limitation found in the analysis of studies exploring the effects of caffeine and coffee on the occurrence of AF is the lack of a comparative analysis between the sexes. The differences between the sexes are important and include genetic (for example the metabolization of caffeine by enzymes), environmental (the differences in the microbiome and microbiota) and lifestyle components (the different way of coping with stress) [31,49,88,89,90,91,92,93]. Few studies have the comparison between men and women as their primary objective, most studies evaluate the population in general or there are studies on exclusively male or female populations [61,73,77].

Another limitation relates to the differences in the source of caffeine between young people and adults. Young people tend to take caffeine through energy drinks or soft drinks. The incidence of atrial fibrillation in young people is very low; however, some case reports illustrate arrhythmic episodes as a consequence of the assumption of high doses of energy drinks [5,94,95,96,97,98,99].

And finally, the effects of caffeine should be evaluated in the overall lifestyle of the subject who develops arrhythmias. Associations between diet, physical activity and sedentary lifestyle and the onset of AF have been reported by several studies [100,101,102].

## 7. Conclusions

The correlation between caffeine and coffee and atrial fibrillation is complex and population studies do not offer unambiguous conclusions. Certainly, there are genetic, environmental and socio-economic influences. The differences between women and men are rarely explored regardless of the conditions for a different response in the two sexes. Comparison studies between women and men are needed to understand which lifestyle modifications are useful for preventing the onset of arrhythmia.

## Figures and Tables

**Table 1:** Levels of caffeine intake according to The Food Standards Authority of Australia and New Zealand.

	Dose (mg/day)	Equivalent to (mg/kg) *
Low intake	80–250	1.1–1.3
Moderate intake:	300–400	4–6
High intake:	>500	7

Legend: * in a 70 kg weight adult.

**Table 2:** European Food Safety Authority (ESFA) dose recommendations for safety problems.

No Safety Problem	Dose (mg/day)	Equivalent to (mg/kg) *
	up to 200 mg	3
Habitual consumers	up to 400 mg	6
Pregnant women habitual consumers	up to 200	3
Breastfeeding women	up to 200	3
Children and adolescent	Unknown	

Legend: * in a 70 kg weight adult.

**Table 3:** Some perspective and cohort studies.

Author [Ref]	Population	Female/Male (%)	Results
Kim et al. [85]	386,258 subjects	52.3/47.7	Each additional cup of habitual coffee consumed was associated with a 3% lower risk of incident arrhythmia (hazard ratio [HR], 0.97; 95% CI, 0.96–0.98; *p* < 0.001).
UK Biobank [20]	449,563 subjects	55.3/44.7	Drinking 4 to 5 cups/day of ground coffee and 2 to 3 cups/day of instant coffee reduced incident arrhythmias
Women Health study [74]	33,638 Subjects Alla females	100/0	No increased risk of AF in women who consumed large amounts of caffeine
Frost [72] Danish Diet, Cancer and Health Study.	47,949 subjects	43.3/56.7	Lowest quintile of caffeine consumption was used as a reference, the adjusted hazard ratios (95% CIs) in quintiles 2: HR 1.12 (0.87, 1.44), quintiles 3: HR 0.85 (0.65, 1.12) quintiles 4: HR 0.92 (0.71, 1.20), quintiles 5: HR 0.91 (0.70, 1.19)
Bodar et al. [77] Physicians’ Health Study	18,960 Subjects (all males)	0/100	The effect of coffee consumption on the risk of AF was dose-related: ≤1 cup/week (NS) 2–4 cups/week (NS) 5–6 cups/week (NS) 1 cup/day (HR = 0.85; 95% CI: 0.74–0.98) 2–3 cups/day (HR = 0.86; 95% CI: 0.76–0.97) • >4 cups/day (NS)
Wilhelmsen et al. [73]	7495 Subjects (all males)	0/100	Consumption of ≥ 5 cups/day was not significantly associated with a higher risk of incident AF, moderate consumption reached borderline significance (OR = 1.24; 95% CI: 1.00–1.54)
Casiglia et al. [78]	1475 subjects	54.6/43.4	Consumption of <320 mg caffeine/day was not significantly associated with the risk reduction in AF, while consumption > 320 mg/day significantly reduced this risk,
Larsson SC et al. [86]	76,475 subjects	41,881 men in the Cohort of Swedish Men and 34,594 women in the Swedish Mammography Cohort	Coffee consumption has not been shown to influence the risk of AF.
Mattioli et al. [48]	247 subjects	45.4/54.6	Consumption of 1 to >3 cups of coffee/day was not significantly associated with spontaneous conversion of AF
Shen et al. [87] Framingham Heart Study	4526 subjects	56/44	Consumption of caffeine was not significantly associated with AF risk. Q1, Q2, Q3 and Q4: NS

## Data Availability

No new data were created or analyzed in this study. Data sharing is not applicable to this article.

## References

[1] Surma S., Romańczyk M., Filipiak K.J., Lip G.Y.H. (2022). Coffee and cardiac arrhythmias: Up-date review of the literature and clinical studies. Cardiol. J..

[2] Sims S.T., Kerksick C.M., Smith-Ryan A.E., Janse de Jonge X.A., Hirsch K.R., Arent S.M., Hewlings S.J., Kleiner S.M., Bustillo E., Tartar J.L. (2023). International society of sports nutrition position stand: Nutritional concerns of the female athlete. J. Int. Soc. Sports Nutr..

[3] Chieng D., Kistler P.M. (2022). Coffee and tea on cardiovascular disease (CVD) prevention. Trends Cardiovasc. Med..

[4] Voskoboinik A., Kalman J.M., Kistler P.M. (2018). Caffeine and Arrhythmias: Time to Grind the Data. JACC Clin. Electrophysiol..

[5] Kaur A., Yousuf H., Ramgobin-Marshall D., Jain R., Jain R. (2022). Energy drink consumption: A rising public health issue. Rev. Cardiovasc. Med..

[6] Mattioli A.V. (2007). Effects of caffeine and coffee consumption on cardiovascular disease and risk factors. Fut. Cardiol..

[7] Khiali S., Agabalazadeh A., Sahrai H., Bannazadeh Baghi H., Rahbari Banaeian G., Entezari-Maleki T. (2023). Effect of Caffeine Consumption on Cardiovascular Disease: An Updated Review. Pharmaceut Med..

[8] Bastian F., Hutabarat O.S., Dirpan A., Nainu F., Harapan H., Emran T.B., Simal-Gandara J. (2021). From Plantation to Cup: Changes in Bioactive Compounds during Coffee Processing. Foods.

[9] Mattioli A.V. (2015). Caffeine and atrial fibrillation. V Preedy Coffee in 1. Health and Disease Prevention.

[10] Food Standards Australian and New Zealand. https://www.foodstandards.gov.au/consumer/generalissues/pages/caffeine.aspx#:~:text=It%20sets%20maximum%20permitted%20levels,is%20320%20mg%20per%20litre.

[11] Standard 2.6.4—Formulated Caffeinated Beverages—Food Standards (Proposal P1025—Code Revision) Variation—Australia New Zealand Food Standards Code—Amendment No. 154. https://gazette.govt.nz/notice/id/2015-gs1907.

[12] (2015). ESFA Journal “Scientific Opinion on the Safety of Caffeine”. https://www.efsa.europa.eu/en/efsajournal/pub/4102.

[13] Kaplan G.B., Greenblatt D.J., Ehrenberg B.L., Goddard J.E., Cotreau M.M., Harmatz J.S., Shader R.I. (1997). Dose-dependent pharmacokinetics and psychomotor effects of caffeine in humans. J. Clin. Pharm..

[14] Lang R., Dieminger N., Beusch A., Lee Y.M., Dunkel A., Suess B., Skurk T., Wahl A., Hauner H., Hofmann T. (2013). Bioappearance and pharmacokinetics of bioactives upon coffee consumption. Anal. Bioanal. Chem..

[15] Mattioli A.V., Manenti A., Farinetti A. (2023). Monitoring Caffeine Intake: The Relevance of Adequate Assessment in the Population. J. Am. Nutr. Assoc..

[16] Ludwig I.A., Mena P., Calani L., Cid C., Del Rio D., Lean M.E., Crozier A. (2014). Variations in caffeine and chlorogenic acid contents of coffees: What are we drinking?. Food Funct..

[17] Mannino G., Kunz R., Maffei M.E. (2023). Discrimination of Green Coffee (Coffea arabica and Coffea canephora) of Different Geographical Origin Based on Antioxidant Activity, High-Throughput Metabolomics, and DNA RFLP Fingerprinting. Antioxidants.

[18] Jeszka-Skowron M., Frankowski R., Zgoła-Grześkowiak A., Płatkiewicz J. (2022). Comprehensive Analysis of Metabolites in Brews Prepared from Naturally and Technologically Treated Coffee Beans. Antioxidants.

[19] Stiefel C., Lindemann B., Morlock G.E. (2022). Non-target bioactive compound profiles of coffee roasts and preparations. Food Chem..

[20] Chieng D., Canovas R., Segan L., Sugumar H., Voskoboinik A., Prabhu S., Ling L.H., Lee G., Morton J.B., Kaye D.M. (2022). The impact of coffee subtypes on incident cardiovascular disease, arrhythmias, and mortality: Long-term outcomes from the UK Biobank. Eur. J. Prev. Cardiol..

[21] Bulczak E.M., Chmurzyńska A.U. (2023). Caffeine Consumption in Polish Adults: Development and Validation of a Polish Questionnaire for Assessing Caffeine Intake. J. Am. Nutr. Assoc..

[22] Sachse C., Brockmöller J., Bauer S., Roots I. (1999). Functional significance of a C→A polymorphism in intron 1 of the cytochrome P450 CYP1A2 gene tested with caffeine. Br. J. Clin. Pharmacol..

[23] Cornelis M.C., El-Sohemy A., Kabagambe E.K., Campos H. (2006). Coffee, CYP1A2 genotype, and risk of myocardial infarction. JAMA.

[24] Palatini P., Benetti E., Mos L., Garavelli G., Mazzer A., Cozzio S., Fania C., Casiglia E. (2015). Association of coffee consumption and CYP1A2 polymorphism with risk of impaired fasting glucose in hypertensive patients. Eur. J. Epidemiol..

[25] Mahdavi S., Palatini P., El-Sohemy A. (2023). CYP1A2 Genetic Variation, Coffee Intake, and Kidney Dysfunction. JAMA Netw. Open.

[26] Huang Y., Shan Y., Zhang W., Lee A.M., Li F., Stranger B.E., Huang R.S. (2023). Deciphering genetic causes for sex differences in human health through drug metabolism and transporter genes. Nat. Commun..

[27] Van Der Weide J., Steijns L.S.W., VanWeelden M.J.M. (2003). The effect of smoking and cytochrome P450 CYP1A2 genetic polymorphism on clozapine clearance and dose requirement. Pharmacogenetics.

[28] Coppi F., Migaldi M., Stefanelli C., Farinetti A., Mattioli A.V. (2023). Changes in coffee and caffeine intake during the pandemic in women smokers and non-smokers: A future challenge for cardiovascular prevention. Acta Biomed..

[29] Casiglia E., Tikhonoff V., Albertini F., Favaro J., Montagnana M., Danese E., Finatti F., Benati M., Mazza A., Dal Maso L. (2017). Caffeine intake and abstract reasoning among 1374 unselected men and women from general population. Role of the -163C>A polymorphism of CYP1A2 gene. Clin. Nutr. ESPEN.

[30] Gkouskou K.G., Georgiopoulos G., Vlastos I., Lazou E., Chaniotis D., Papaioannou T.G., Mantzoros C.S., Sanoudou D., Eliopoulos A.G. (2022). CYP1A2 polymorphisms modify the association of habitual coffee consumption with appetite, macronutrient intake, and body mass index: Results from an observational cohort and a cross-over randomized study. Int. J. Obes..

[31] Mattioli A.V., Moscucci F., Sciomer S., Maffei S., Nasi M., Pinti M., Bucciarelli V., Dei Cas A., Parati G., Ciccone M. (2023). Cardiovascular prevention in women: Un update By the Italian Society of Cardiology Working Group on “Prevention, Hypertension and peripheral disease”. J. Cardiovasc. Med..

[32] Cornelis M.C., Kacprowski T., Menni C., Gustafsson S., Pivin E., Adamski J., Artati A., Eap C.B., Ehret G., Friedrich N. (2016). Genome-wide association study of caffeine metabolites provides new insights to caffeine metabolism and dietary caffeine-consumption behavior. Hum. Mol. Genet..

[33] Fredholm B.B., Battic K., Holmen J., Nehlig A., Zvartau E.E. (1999). Actions of 2. caffeine in the brain with special reference to factors that contribute to its widespread use. Pharmacol. Rev..

[34] Röhrig T., Liesenfeld D., Richling E. (2017). Identification of a Phosphodiesterase-Inhibiting Fraction from Roasted Coffee (Coffea arabica) through Activity-Guided Fractionation. J. Agric. Food Chem..

[35] Barone J.J., Roberts H.R. (1996). Caffeine consumption. Food Chem. Toxicol..

[36] Bolignano D., Coppolino G., Barillà A., Campo S., Criseo M., Tripodo D., Buemi M. (2007). Caffeine and the kidney: What evidence right now?. J. Ren. Nutr..

[37] Echeverri D., Montes F.R., Cabrera M., Galán A., Prieto A. (2010). Caffeine’s Vascular Mechanisms of Action. Int. J. Vasc. Med..

[38] Zhang L., Yin J., Li J., Sun H., Liu Y., Yang J. (2023). Association between dietary caffeine intake and severe headache or migraine in US adults. Sci Rep..

[39] Bucciarelli V., Mattioli A.V., Sciomer S., Moscucci F., Renda G., Gallina S. (2023). The Impact of Physical Activity and Inactivity on Cardiovascular Risk across Women’s Lifespan: An Updated Review. J. Clin. Med..

[40] Susy K. (2023). Long-term outcomes from the UK Biobank on the impact of coffee on cardiovascular disease, arrhythmias, and mortality: Does the future hold coffee prescriptions?. Glob. Cardiol. Sci. Pract..

[41] Marcus G.M., Modrow M.F., Schmid C.H., Sigona K., Nah G., Yang J., Chu T.C., Joyce S., Gettabecha S., Ogomori K. (2022). Individualized Studies of Triggers of Paroxysmal Atrial Fibrillation: The I-STOP-AFib Randomized Clinical Trial. JAMA Cardiol..

[42] Mattioli A.V. (2019). Link between coffee and atrial fibrillation debunked?. Expert. Rev. Cardiovasc. Ther..

[43] Borghi C. (2022). Coffee and blood pressure: Exciting news!. Blood Press..

[44] Mattioli A.V., Selleri V., Zanini G., Nasi M., Pinti M., Stefanelli C., Fedele F., Gallina S. (2022). Physical Activity and Diet in Older Women: A Narrative Review. J. Clin. Med..

[45] Guieu R., Degioanni C., Fromonot J., De Maria L., Ruf J., Deharo J.C., Brignole M. (2022). Adenosine, Adenosine Receptors and Neurohumoral Syncope: From Molecular Basis to Personalized Treatment. Biomedicines.

[46] O’Keefe J.H., Bhatti S.K., Patil H.R., DiNicolantonio J.J., Lucan S.C., Lavie C.J. (2013). Effects of habitual coffee consumption on cardiometabolic disease, cardiovascular health, and all-cause mortality. J. Am. Coll. Cardiol..

[47] Ochoa-Rosales C., van der Schaft N., Braun K.V.E., Ho F.K., Petermann-Rocha F., Ahmadizar F., Kavousi M., Pell J.P., Ikram M.A., Celis-Morales C.A. (2023). C-reactive protein partially mediates the inverse association between coffee consumption and risk of type 2 diabetes: The UK Biobank and the Rotterdam study cohorts. Clin. Nutr..

[48] Mattioli A.V., Farinetti A., Miloro C., Pedrazzi P., Mattioli G. (2011). Influence of coffee and caffeine consumption on atrial fibrillation in hypertensive patients. Nutr. Metab. Cardiovasc. Dis..

[49] Machado F., Coimbra M.A., Castillo M.D.D., Coreta-Gomes F. (2023). Mechanisms of action of coffee bioactive compounds—A key to unveil the coffee paradox. Crit. Rev. Food Sci. Nutr..

[50] Costa V.M., Grando L.G.R., Milandri E., Nardi J., Teixeira P., Mladěnka P., Remião F. (2022). On Behalf Of The Oemonom. Natural Sympathomimetic Drugs: From Pharmacology to Toxicology. Biomolecules.

[51] Cicero A.F., Fogacci F., D’Addato S., Grandi E., Rizzoli E., Borghi C., Brisighella Heart Study (2023). Self-Reported Coffee Consumption and Central and Peripheral Blood Pressure in the Cohort of the Brisighella Heart Study. Nutrients.

[52] Marcus G.M., Rosenthal D.G., Nah G., Vittinghoff E., Fang C., Ogomori K., Joyce S., Yilmaz D., Yang V., Kessedjian T. (2023). Acute Effects of Coffee Consumption on Health among Ambulatory Adults. N. Engl. J. Med..

[53] Machado M., Espírito Santo L., Machado S., Lobo J.C., Costa A.S., Oliveira M.B.P., Ferreira H., Alves R.C. (2023). Bioactive Potential and Chemical Composition of Coffee By-Products: From Pulp to Silverskin. Foods.

[54] Lemos M.F., Salustriano N.A., Costa M.M.S., Lirio K., Fonseca A.F.A., Pacheco H.P., Endringer D.C., Fronza M., Scherer R. (2022). Chlorogenic acid and caffeine contents and anti-inflammatory and antioxidant activities of green beans of conilon and arabica coffees harvested with different degrees of maturation. J. Saudi. Chem. Soc..

[55] Mattioli A.V., Francesca C., Mario M., Farinetti A. (2018). Fruit and vegetables in hypertensive women with asymptomatic peripheral arterial disease. Clin. Nutr. ESPEN.

[56] Cyr A.R., Huckaby L.V., Shiva S.S., Zuckerbraun B.S. (2020). Nitric Oxide and Endothelial Dysfunction. Crit. Care Clin..

[57] Lu M.Y., Lai J.C., Chen S.J. (2023). Influence of Sex Differences on Serum Lipid Profiles among Habitual Coffee Drinkers: Evidence from 23,072 Taiwan Biobank Participants. Nutrients.

[58] Tan X., Sun Y., Chen L., Hu J., Meng Y., Yuan M., Wang Q., Li S., Zheng G., Qiu Z. (2022). Caffeine Ameliorates AKT-Driven Nonalcoholic Steatohepatitis by Suppressing De Novo Lipogenesis and MyD88 Palmitoylation. J. Agric. Food Chem..

[59] Mattioli A.V., Migaldi M., Farinetti A. (2018). Coffee in hypertensive women with asymptomatic peripheral arterial disease: A potential nutraceutical effect. J. Cardiovasc. Med..

[60] Vandenberghe C., St-Pierre V., Courchesne-Loyer A., Hennebelle M., Castellano C.A., Cunnane S.C. (2017). Caffeine intake increases plasma ketones: An acute metabolic study in humans. Can. J. Physiol. Pharmacol..

[61] Kim J., Park J., Lim K. (2016). Nutrition Supplements to Stimulate Lipolysis: A Review in Relation to Endurance Exercise Capacity. J. Nutr. Sci. Vitaminol..

[62] Yamada A.K., Pimentel G.D., Pickering C., Cordeiro A.V., Silva V.R.R. (2022). Effect of caffeine on mitochondrial biogenesis in the skeletal muscle—A narrative review. Clin. Nutr. ESPEN.

[63] Schnuck J.K., Gould L.M., Parry H.A., Johnson M.A., Gannon N.P., Sunderland K.L., Vaughan R.A. (2018). Metabolic effects of physiological levels of caffeine in myotubes. J. Physiol. Biochem..

[64] Mattioli A.V. (2022). Coffee consumption effects on bioelectrical impedance parameters: Does gender matter?. Eur. J. Clin. Nutr..

[65] Slighoua M., Amrati F.E.Z., Chebaibi M., Mahdi I., Al Kamaly O., El Ouahdani K., Drioiche A., Saleh A., Bousta D. (2023). Quercetin and Ferulic Acid Elicit Estrogenic Activities In Vivo and In Silico. Molecules.

[66] Gohil K.J., Kshirsagar S.B., Sahane R.S. (2012). Ferulic Acid-A Comprehensive Pharmacology of an Important Bioflavonoid. Int. J. Pharm. Sci. Res..

[67] Krause D.N., Duckles S.P., Pelligrino D.A. (2006). Influence of Sex Steroid Hormones on Cerebrovascular Function. J. Appl. Physiol..

[68] Surma S., Sahebkar A., Banach M. (2023). Coffee or tea: Anti-inflammatory properties in the context of atherosclerotic cardiovascular disease prevention. Pharmacol. Res..

[69] Ren Y., Wang C., Xu J., Wang S. (2019). Cafestol and Kahweol: A Review on Their Bioactivities and Pharmacological Properties. Int. J. Mol. Sci..

[70] de Roos B., Caslake M.J., Stalenhoef A.F., Bedford D., Demacker P.N., Katan M.B., Packard C.J. (2001). The coffee diterpene cafestol increases plasma triacylglycerol by increasing the production rate of large VLDL apolipoprotein B in healthy normolipidemic subjects. Am. J. Clin. Nutr..

[71] Coelho M., Patarrão R.S., Sousa-Lima I., Ribeiro R.T., Meneses M.J., Andrade R., Mendes V.M., Manadas B., Raposo J.F., Macedo M.P. (2022). Increased Intake of Both Caffeine and Non-Caffeine Coffee Components Is Associated with Reduced NAFLD Severity in Subjects with Type 2 Diabetes. Nutrients.

[72] Frost L., Vestergaard P. (2005). Caffeine and risk of atrial fibrillation or flutter: The Danish Diet, Cancer, and Health Study. Am. J. Clin. Nutr..

[73] Wilhelmsen L., Rosengren A., Lappas G. (2001). Hospitalizations for atrial fibrillation in the general male population: Morbidity and risk factors. J. Intern. Med..

[74] Conen D., Chiuve S.E., Everett B.M., Zhang S.M., Buring J.E., Albert C.M. (2010). Caffeine consumption and incident atrial fibrillation in women. Am. J. Clin. Nutr..

[75] Mattioli A.V., Tarabini Castellani E., Vivoli D., Molinari R., Mattioli G. (1996). Restoration of atrial function after atrial fibrillation of different etiological origins. Cardiology.

[76] Yuan S., Larsson S.C. (2019). No association between coffee consumption and risk of atrial fibrillation: A Mendelian randomization study. Nutr. Metab. Cardiovasc. Dis..

[77] Bodar V., Chen J., Gaziano J.M., Albert C., Djoussé L. (2019). Coffee consumption and risk of atrial fibrillation in the Physicians’ Health Study. J. Am. Heart Assoc..

[78] Casiglia E., Tikhonoff V., Albertini F., Gasparotti F., Mazza A., Montagnana M., Danese E., Benati M., Spinella P., Palatini P. (2018). Caffeine intake reduces incident atrial fibrillation at a population level. Eur. J. Prev. Cardiol..

[79] Mattioli A.V., Bonatti S., Zennaro M., Melotti R., Mattioli G. (2008). Effect of coffee consumption, lifestyle and acute life stress in the development of acute lone atrial fibrillation. J. Cardiovasc. Med..

[80] Leo D.G., Ozdemir H., Lane D.A., Lip G.Y.H., Keller S.S., Proietti R. (2023). At the heart of the matter: How mental stress and negative emotions affect atrial fibrillation. Front. Cardiovasc. Med..

[81] Mattioli A.V., Coppi F., Nasi M., Gallina S. (2022). Stress and cardiovascular risk burden after the pandemic: Current status and future prospects. Expert. Rev. Cardiovasc. Ther..

[82] Jakobsdottir G., Stefansdottir R.S., Gestsdottir S., Stefansson V., Johannsson E., Rognvaldsdottir V., Gisladottir T.L. (2023). Changes in health-related lifestyle choices of university students before and during the COVID-19 pandemic: Associations between food choices, physical activity and health. PLoS ONE.

[83] Adcock S., Lang B. (2023). Caffeine Motives and Expectancies for Individuals with High Anxiety Sensitivity. Subst. Use Misuse..

[84] Mattioli A.V., Sabatini S. (2021). Changes in energy drink consumption during the COVID-19 quarantine. Clin. Nutr. ESPEN.

[85] Kim E.J., Hoffmann T.J., Nah G., Vittinghoff E., Delling F., Marcus G.M. (2021). Coffee consumption and incident tachyarrhythmias: Reported behavior, Mendelian randomization, and their interactions. JAMA Intern. Med..

[86] Larsson S.C., Drca N., Jensen-Urstad M., Wolk A. (2015). Coffee consumption is not associated with increased risk of atrial fibrillation: Results from two prospective cohorts and a meta-analysis. BMC Med..

[87] Shen J., Johnson V.M., Sullivan L.M., Jacques P.F., Magnani J.W., Lubitz S.A., Pandey S., Levy D., Vasan R.S., Quatromoni P.A. (2011). Dietary factors and incident atrial fibrillation: The Framingham Heart Study. Am. J. Clin. Nutr..

[88] Kapellou A., King A., Graham C.A.M., Pilic L., Mavrommatis Y. (2023). Genetics of caffeine and brain-related outcomes—A systematic review of observational studies and randomized trials. Nutr. Rev..

[89] Mostofsky E., Berg Johansen M., Tjønneland A., Chahal H.S., Mittleman M.A., Overvad K. (2017). Chocolate intake and risk of clinically apparent atrial fibrillation: The Danish Diet, Cancer, and Health Study. Heart.

[90] Li Y., Xu Y., Le Roy C., Hu J., Steves C.J., Bell J.T., Spector T.D., Gibson R., Menni C., Rodriguez-Mateos A. (2023). Interplay between the (Poly)phenol Metabolome, Gut Microbiome, and Cardiovascular Health in Women: A Cross-Sectional Study from the Twins UK Cohort. Nutrients.

[91] Dai A., Hoffman K., Xu A.A., Gurwara S., White D.L., Kanwal F., Jang A., El-Serag H.B., Petrosino J.F., Jiao L. (2023). The Association between Caffeine Intake and the Colonic Mucosa-Associated Gut Microbiota in Humans-A Preliminary Investigation. Nutrients.

[92] Salerni S., Di Francescomarino S., Cadeddu C., Acquistapace F., Maffei S., Gallina S. (2015). The different role of sex hormones on female cardiovascular physiology and function: Not only oestrogens. Eur. J. Clin. Investig..

[93] Cadeddu C., Franconi F., Cassisa L., Campesi I., Pepe A., Cugusi L., Maffei S., Gallina S., Sciomer S., Mercuro, G., on behalf of the Working Group of Gender Medicine of Italian Society of Cardiology (2016). Arterial hypertension in the female world: Pathophysiology and therapy. J. Cardiovasc. Med..

[94] Domaszewski P. (2023). Gender Differences in the Frequency of Positive and Negative Effects after Acute Caffeine Consumption. Nutrients.

[95] Mattioli A.V., Pennella S., Farinetti A., Manenti A. (2018). Energy Drinks and atrial fibrillation in young adults. Clin. Nutr..

[96] Ellermann C., Hakenes T., Wolfes J., Wegner F.K., Willy K., Leitz P., Rath B., Eckardt L., Frommeyer G. (2022). Cardiovascular risk of energy drinks: Caffeine and taurine facilitate ventricular arrhythmias in a sensitive whole-heart model. J. Cardiovasc. Electrophysiol..

[97] Lévy S., Cappato R. (2022). Cardiovascular Adverse Events Associated with Energy Drinks in Adolescents and Young Adults. Cardiovasc. Drugs Ther..

[98] Coppi F., Nasi M., Farinetti A., Manenti A., Sabina G., Mattioli A.V. (2021). Physical activity, sedentary behaviour, and diet in menopausal women: Comparison between COVID19 “first wave” and “second wave” of pandemic in Italy. Prog. Nutr..

[99] Yang L., Chung M.K. (2023). Lifestyle changes in atrial fibrillation management and intervention. J. Cardiovasc. Electrophysiol.

[100] Truzzi M.L., Puviani M.B., Tripodi A., Toni S., Farinetti A., Nasi M., Mattioli A.V. (2020). Mediterranean Diet as a model of sustainable, resilient and healthy diet. Prog. Nutr..

[101] Gupta V., Munjal J.S., Jhajj P., Jhajj S., Jain R. (2022). Obesity and Atrial Fibrillation: A Narrative Review. Cureus.

[102] Bizhanov K.A., Abzaliyev K.B., Baimbetov A.K., Sarsenbayeva A.B., Lyan E. (2023). Atrial fibrillation: Epidemiology, pathophysiology, and clinical complications (literature review). J. Cardiovasc. Electrophysiol..

